# Supplementary material for: MetaQTL: a package of new computational methods for the meta-analysis of QTL mapping experiments
Source: BMC Bioinformatics. 2007 Feb 8;8:49. doi: 10.1186/1471-2105-8-49 (PMC1808479; doi:10.1186/1471-2105-8-49)
Supplement: Additional File 7 — MetaQTL Package : jar file and tutorial. This Zip archive contains both the MetaQTL JAR file and the files of the tutorial. [file 1471-2105-8-49-S7.zip › org.inra.metaqtl/doc/serialized-form.html]

Serialized Form


|  |  |  |
| --- | --- | --- |
| **Overview** \| Package \| Class \| Use \| **Tree** \| **Deprecated** \| **Index** \| **Help** | |  |
| PREV   NEXT | **FRAMES**    **NO FRAMES**     **All Classes** |  |


---


# Serialized Form


---

**Package** **org.metaqtl**

**Class org.metaqtl.EMCriteria extends java.lang.Object implements Serializable**

**serialVersionUID:** 1L

**Serialized Fields**

### aic

```
double aic
```

---

### aicc

```
double aicc
```

---

### aic3

```
double aic3
```

---

### bic

```
double bic
```

---

### mir

```
double mir
```

---

### icomp

```
double icomp
```

---

### awe

```
double awe
```

**Class org.metaqtl.Qtl.QTLCross extends java.lang.Object implements Serializable**

**serialVersionUID:** 1L

**Serialized Fields**

### name

```
java.lang.String name
```

:   The name of the cross design.

---

### type

```
int type
```

:   The type of the cross.

---

### gen

```
int gen
```

:   The number of generations.

---

### size

```
int size
```

:   The number of individuals.

**Class org.metaqtl.Qtl.QTLProj extends java.lang.Object implements Serializable**

**serialVersionUID:** 1L

**Serialized Fields**

### mapOrigin

```
java.lang.String mapOrigin
```

:   The name of the map from which
    this QTL comes.

---

### o\_mrkIdxL

```
int o_mrkIdxL
```

:   The original indice of the left
    flanking marker.

---

### o\_mrkIdxR

```
int o_mrkIdxR
```

:   The original indice of the right
    flanking marker.

---

### s\_mrkIdxL

```
int s_mrkIdxL
```

:   The skeleton indice of the left
    flanking marker.

---

### s\_mrkIdxR

```
int s_mrkIdxR
```

:   The skeleton indice of the right
    flanking marker.

---

### mapScale

```
double mapScale
```

:   The scale ratio between the original
    map and the map on which the QTL has
    been projected.

---

### mapWeight

```
double mapWeight
```

:   The cumulated scale weights of the common
    marke sequences between the original map
    and the map on which the QTL has been projected.

---

### intScale

```
double intScale
```

:   The scale ratio between the original flanking
    marker interval and the one on the map used
    for projection.

---

### shareFlanking

```
boolean shareFlanking
```

:   True if the two maps share a common marker sequence
    around the QTL.

---

### swap

```
boolean swap
```

:   True if the flanking marker order are inversed between
    the skeleton and the original map.

**Class org.metaqtl.Qtl.QTLStats extends java.lang.Object implements Serializable**

**serialVersionUID:** 1L

**Serialized Fields**

### rsquare

```
double rsquare
```

:   The r-square, i.e the proportion of variance
    explained by the QTL.

---

### additive

```
double additive
```

:   The additive effect of the QTL.

---

### dominance

```
double dominance
```

:   The dominance effect of the QTL.

---

### lod

```
double lod
```

:   The lod score of the QTL.

**Class org.metaqtl.Qtl.QTLSupport extends java.lang.Object implements Serializable**

**serialVersionUID:** 1L

**Serialized Fields**

### ic1

```
double ic1
```

:   The recombination rate between the QTL position
    and the left border of the IC.

---

### ic2

```
double ic2
```

:   The recombination rate between the QTL position
    and the right border of the IC.

---

### from

```
double from
```

:   The position from the first marker
    to the left border of the IC.

---

### to

```
double to
```

:   The position from the first marker
    to the right border of the IC.

---

### lodDecrease

```
double lodDecrease
```

:   The value of the LOD decrease used to obtain the CI.

**Class org.metaqtl.Qtl.QTLTrait extends java.lang.Object implements Serializable**

**serialVersionUID:** 1L

**Serialized Fields**

### name

```
java.lang.String name
```

:   The name of the trait.

---

### unit

```
java.lang.String unit
```

:   The unit of the trait.

---

### group

```
java.lang.String group
```

:   The name of the group to which
    the trait belongs.

**Class org.metaqtl.TreeNode extends java.lang.Object implements Serializable**

**serialVersionUID:** 1L

**Serialized Fields**

### idx

```
int idx
```

:   ?
    The identifiant of the cluster.

---

### label

```
java.lang.String label
```

:   The label of the cluster

---

### card

```
int card
```

:   The number of sequences in the cluster

---

### nidx

```
int[] nidx
```

:   The indices of the sub-nodes

---

### leaf

```
boolean leaf
```

:   The status of the node.

---

### dist

```
double dist
```

:   The distance between the node and its parent.

---

### height

```
double height
```

:   The distance between the node and the root of the tree (if defined).

---

### children

```
TreeNode[] children
```

:   Left and right children, or null

---

### parent

```
TreeNode parent
```

:   The parent of the node

---

### x

```
double x
```

---

### y

```
double y
```

---

### xc

```
double xc
```

---

**Package** **org.metaqtl.bio.entity**

**Class org.metaqtl.bio.entity.BioEntityCollection extends java.util.ArrayList implements Serializable**

**serialVersionUID:** 1L

**Serialized Fields**

### name

```
java.lang.String name
```

---

### parent

```
IBioEntity parent
```

---

### properties

```
java.util.Properties properties
```

---

**Package** **org.metaqtl.main**

**Class org.metaqtl.main.CmdLineParser.IllegalOptionValueException extends CmdLineParser.OptionException implements Serializable**

**Serialized Fields**

### option

```
CmdLineParser.Option option
```

---

### value

```
java.lang.String value
```

**Class org.metaqtl.main.CmdLineParser.NotFlagException extends CmdLineParser.UnknownOptionException implements Serializable**

**Serialized Fields**

### notflag

```
char notflag
```

**Class org.metaqtl.main.CmdLineParser.OptionException extends java.lang.Exception implements Serializable**

**Class org.metaqtl.main.CmdLineParser.UnknownOptionException extends CmdLineParser.OptionException implements Serializable**

**Serialized Fields**

### optionName

```
java.lang.String optionName
```

**Class org.metaqtl.main.CmdLineParser.UnknownSuboptionException extends CmdLineParser.UnknownOptionException implements Serializable**

**Serialized Fields**

### suboption

```
char suboption
```

---


|  |  |  |
| --- | --- | --- |
| **Overview** \| Package \| Class \| Use \| **Tree** \| **Deprecated** \| **Index** \| **Help** | |  |
| PREV   NEXT | **FRAMES**    **NO FRAMES**     **All Classes** |  |


---
